# The Effects of COVID‐19 on Tinnitus Severity and Quality of Life in Individuals With Subjective Tinnitus

**DOI:** 10.1002/brb3.70317

**Published:** 2025-02-19

**Authors:** Zehra Aydogan, Mehmet Can, Emre Soylemez, Kursad Karakoc, Zahide Ciler Buyukatalay, Suna Tokgoz Yilmaz

**Affiliations:** ^1^ Department of Audiology Faculty of Health Sciences Ankara University Ankara Turkey; ^2^ Audiometry Program, Vocational School of Health Services Karaman University Karaman Turkey; ^3^ Audiometry Program, Vocational School of Health Services Karabuk University Karabuk Turkey; ^4^ Department of Audiology, Faculty of Health Sciences Ankara Yıldırım Beyazıt University Ankara Turkey; ^5^ Ibn‐i Sina Hospital, Otolaryngology Department, Faculty of Medicine Ankara University Ankara Turkey

**Keywords:** COVID‐19, tinnitus, tinnitus severity, quality of life

## Abstract

**Purpose:**

This study aimed to investigate the effects of the SARS‐CoV‐2 virus (COVID‐19) on tinnitus severity and quality of life in infected and non‐infected individuals who were re‐admitted to our clinic after the COVID‐19 outbreak.

**Methods:**

The study involved both retrospective and prospective data analysis. The study included 30 individuals aged 20–65 years with normal hearing who had undergone psychoacoustic, tinnitus, and psychosomatic evaluations before the pandemic. Participants were divided into Group 1 (*n* = 15, COVID‐19 negative) and Group 2 (*n* = 15, COVID‐19 positive). Before the pandemic, tinnitus‐related evaluations of all participants (i.e., pure tone audiometry [PTA], tinnitus frequency, loudness, minimum masking level [MML], and residual inhibition [RI]) were conducted, and the Tinnitus Handicap Inventory (THI), Visual Analog Scale (VAS), and Short Form 36 (SF‐36) were administered to all participants. All assessments were repeated after the outbreak of the pandemic.

**Results:**

There was no significant difference in the mean PTA thresholds of both groups before and after the pandemic (*p* > 0.05). There was a significant increase in tinnitus severity after COVID‐19 infection in Group 2 (*p* < 0.05). Moreover, it was found that the VAS (loudness and annoyance), THI (functional, emotional, catastrophic, and total score), and SF‐36 (physical function, physical role, pain, general health, vitality, emotional role, and social function) values worsened significantly compared to before COVID‐19 infection (*p* < 0.05). In Group 1, the only significant differences were found in the THI emotional subdomain and the SF‐36 emotional and general health subdomains (*p* < 0.05). No significant differences were found in the other evaluations of Group 1, neither before nor after the pandemic (*p* > 0.05).

**Conclusion:**

Although negative effects of the pandemic were observed in individuals with tinnitus who were not infected with COVID‐19, both the quality of life and tinnitus severity of individuals with tinnitus who were infected with COVID‐19 worsened.

## Graphical abstract

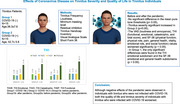

## 1 Introduction

Since December 2019, the world has been in a delicate situation with regard to the health of the population, and the effects of this situation continue to be observed. SARS‐CoV‐2 (COVID‐19) is an infectious disease that spreads rapidly and affects much of the world's population (World Health Organization 2023). Many measures have been taken to reduce this spread, like social distancing, quarantine, and vaccination. In turn, much of society was affected by problems, including anxiety, stress, and psychological disorders related to the pandemic (Özdin and Özdin 2020; Kose et al. 2022).

Tinnitus, a subjective symptom described in the literature as the perception of a “phantom” sound, is a common condition characterized by the detection of a sound in the absence of a corresponding stimulus. Tinnitus is often associated with emotional distress, depressive symptoms, anxiety, and insomnia. Moreover, tinnitus is clinically heterogeneous in terms of its cause, perceptual features, and accompanying symptoms (World Health Organization 2023; Özdin and Özdin 2020; Eggermont and Roberts 2004; Henry et al. 2014).

There is recent evidence that COVID‐19 may be a trigger for problems in the auditory and vestibular systems (Özdin and Özdin 2020; Ahmed et al. 2017). At the same time, approximately 10% of COVID‐19 patients reported new‐onset tinnitus, while existing ones have been reported to increase in severity (Ahmed et al. 2017; Andersson and McKenna 2006; Lough et al. 2022). In addition to tinnitus, COVID‐19 causes many symptoms and profoundly affects individuals' lives.

Our aim was to investigate the effects of COVID‐19 on the severity of tinnitus and quality of life in individuals who were re‐admitted to our clinic after the COVID‐19 pandemic broke out, including individuals who were both infected and not infected with COVID‐19. In our clinic, audiological evaluation and tinnitus psychoacoustic–psychosomatic evaluation are performed on a standard basis for individuals with tinnitus complaints. Understanding the consequences of the virus‐induced changes in symptoms, such as tinnitus and pain, is important for clinicians in this field.

## 2 Materials and Methods

The study is based on data collected between January 2018 and March 2020 at the ear, nose, and throat medical school and audiology center. The approval of the Ethics Board (Decision No: 2022‐02‐28T00_04_39, November 12, 2020, Ethics Board for Human Studies, Medical School, Ankara University) and the approval of the Ministry of Health (Approval Code: 2022‐02‐28T00_04_39) were obtained. Data that met the inclusion criteria were retrieved from the archive. The Declaration of Helsinki principles were followed and informed consent was obtained from all participants before the study.

### 2.1 Participants

Individuals who came to our clinic with a complaint of tinnitus before the pandemic of COVID‐19 had routine/standard audiological evaluation, and tinnitus psychoacoustic–psychosomatic evaluation in our clinic were included in the study. Data regarding individuals who met the inclusion criteria for the study were obtained from the archive. According to the data, patients with subjective tinnitus were called by phone and invited to the hospital. A total of 30 individuals (16 females and 14 males) aged 20–65 years were included in the study, all of whom applied to the ENT department with a complaint of tinnitus (Table 1). Patients were asked whether they had COVID‐19, and standard audiological evaluation and tinnitus psychoacoustic evaluations were repeated. Patients who were officially diagnosed with COVID‐19 (positive [+] polymerase chain reaction [PCR]) and those who were not (negative [−] PCR) were included in the study.

**TABLE 1:** General characteristics of participants (demographic information).

	Group 1	Group 2	*p*
Gender (male/female %)	46.7%–53.3%	60%–40%	
Age (years)	38.33 ± 7.65	42.73 ± 5.87	0.089
Tinnitus duration (years)	5.2 ± 1.2	5.6 ± 2.3	0.135
Tinnitus localization (%)			
Unilateral (right)	20	26.6	
Unilateral (left)	26.6	33.3	
Bilateral	53.3	40	
Tinnitus sound type (%)			
Ringing	46.6	53.3	
Breeze	26.6	20	
Other	26.6	26.6	
Education (%)			
Primary school	13.3	20	
Secondary‐high school	46.6	46.6	
University +	40	33.3	

COVID‐19 severity level (Group 2)			
	Number of person (*n*)	Hospital admission history	
Light level	8	None	
Medium level	5	None	
Severe level	2	None	

*Note*: Data has presented as mean ± standard deviation or percentage. Fifteen of the 30 patients in the study did not have COVID‐19 (Group 1), and the remaining 15 had COVID‐19 within 6 months. The mean age of Group 1 was 38.33 ± 7.65. In Group 2, the mean age was 42.73 ± 5.87 years. The mean time since tinnitus onset was 5.2 ± 1.2 years in Group 1 and 5.6 ± 2.3 years in Group 2. Tinnitus was bilateral in all groups in terms of localization. In terms of tinnitus sound features, individuals in both groups reported a high rate of ringing. The severity of COVID‐19 (Group 2) was classified into three groups: mild, moderate, and severe, with the number of individuals in each group being 8, 5, and 2, respectively.

The inclusion criteria were normal otoscopic results, normal hearing (pure‐tone hearing thresholds of less than 20 dB at 0.5, 1, 2, and 4 kHz and an air‐bone gap of less than 10 dB HL), normal tympanogram (with a Type A), normal limits of ipsilateral and contralateral acoustic reflex thresholds (at 0.5, 1, 2, and 4 kHz), presence of pre‐pandemic audiological evaluation and tinnitus psychoacoustic evaluation, for patients included in Group 2, having received a positive PCR test within 6 months, and no history of systemic diseases.

The exclusion criteria were; patients were excluded from the study if they did not comply with the inclusion criteria. Patients who have not had a PCR test before (unspecified patients).

### 2.2 Procedure

The pure tone audiometry (PTA), acoustic immittance assessment, and psychoacoustic evaluation of tinnitus were performed using the AC‐40 clinical audiometer (Interacoustics, Denmark). For the psychoacoustic evaluation of tinnitus, measurements included tinnitus intensity (dB), frequency (Hz), minimum masking level (MML), and residual inhibition (RI).

In unilateral tinnitus cases, tinnitus frequency and intensity were determined using the contralateral ear. This approach was chosen because patients typically found it easier to match their tinnitus from the contralateral side, allowing for more accurate perception and measurement of intensity. In bilateral tinnitus cases, measurements were conducted for both ears, with the results averaged to provide a comprehensive evaluation.

MML was measured from the ipsilateral ear. RI testing involved applying an ipsilateral pure tone or noise at the tinnitus frequency and 10 dB above the MML for 60 s. Following this, patients were asked whether they experienced a reduction or disappearance of their tinnitus.

The Visual Analog Scale (VAS) and Tinnitus Handicap Inventory (THI) were also used to measure tinnitus severity and discomfort. The Short Form 36 (SF‐36) Quality of Life Scale was used to measure the effects of tinnitus on quality of life.

### 2.3 Tinnitus‐Demographic Data Form

The contributors have compiled this for information purposes. This consisted of questions including demographic information, such as age, sex/gender, level of education, smoking/drinking of alcohol, time of onset and duration of tinnitus, type of tinnitus, treatment/therapy of tinnitus, hearing loss, buzzing in the ear, and feeling of fullness. In addition, participants who had contracted COVID‐19 were asked about the severity of their illness, categorized as mild, moderate, or severe (Table 1). The form's questions were asked verbally by the clinician in accordance with the applicable social distancing rules.

### 2.4 VAS

The severity of the tinnitus and the degree of discomfort from the tinnitus were questioned using the VAS to psychometrically assess the symptoms. Individuals were asked to give a score from 0 to 10 for the severity of the tinnitus and the degree of discomfort from the tinnitus, with 0 meaning “I have no tinnitus, I am not bothered at all” and 10 meaning “I have severe tinnitus, I am very uncomfortable.” The larger the deviation from zero, the greater the resonance intensity (loudness) and the greater the disturbance.

### 2.5 THI

The THI is one of the most common scales for the assessment of tinnitus severity (Andersson and McKenna 2006). The THI is easy to administer and provides specific psychometric measures. This consists of 25 questions answered with “yes” (4 points), “sometimes” (2 points), or “no” (0 points). The total THI score ranges from 0 to 100, and the higher the THI score, the greater the tinnitus‐related handicap (Wilson et al. 1991). THI has subdomains, including emotional, functional, and catastrophic, and the tinnitus level is determined by the total THI score (Levels 1–5). The valid and reliable Turkish version of THI was used (Aksoy et al. 2007).

### 2.6 SF‐36 Quality of Life Scale

The quality of life of the participants was assessed using this form. This scale includes 36 items that evaluate 8 subdomains (physical function [PF], limitation‐physical role [PR], limitation‐emotional role [EP], energy/fatigue [E/F], emotional well‐being [E], social function [SF], pain [P], and general health [GH] perception). Scores for each subscale range from 0 to 100. The more highly the score of each subdomain is scored, the better the health‐related quality of life is assessed (Kocyigit et al. 1999). Turkish validation and reliability of SF‐36 were used.

### 2.7 Statistical Analysis

Power was estimated using G‐Power, and power was estimated at 95% (*α* = 0.05, effect size = 0.85). Results were assessed using the Statistical Package for the Social Sciences (SPSS) version 26.0 (IBM, USA) software. Categorical data are presented as number (*n*) and percentage (%), and descriptive data are presented as means ± standard deviations or medians (interquartile range [IQR]). Histograms and bell curves were used to assess whether the data followed a normal distribution. For comparisons between groups that were not normally distributed, the Mann–Whitney *U* test was used. The paired *t*‐test was used for between‐group variances when the data were normally distributed, and the Wilcoxon ranked test was used when the data were not normally distributed. Results were analyzed using a 95% confidence interval (CI) and a significance level of *p* < 0.05 for all analyses.

## 3 Results

Of the 30 patients in the study, 15 did not have COVID‐19 (Group 1), and the remaining 15 had COVID‐19 within 6 months. The mean age of Group 1 was 38.33 ± 7.65. In Group 2, the mean age was 42.73 ± 5.87 years. The mean time since tinnitus onset was 5.2 ± 1.2 years in Group 1 and 5.6 ± 2.3 years in Group 2. Tinnitus was bilateral in all groups in terms of localization. In terms of tinnitus sound features, individuals in both groups reported a high rate of ringing (Table 1).

There was no significant difference in the mean PTA thresholds of both groups before and after the pandemic (*p* = 0.094, *p* = 0.056). Tinnitus severity (−12.54 to −6.12), MML (−10.19 to −1.40), VAS severity (−2.72 to −1.67), and VAS annoyance (−2.86 to −0.59) of Group 2 worsened after COVID‐19, compared to before COVID‐19 (Table 2). Tinnitus severity (*p* = 0.047), VAS severity (*p* = 0.007), and VAS annoyance (*p* = 0.009) in patients who had COVID‐19 were worse than in patients who did not have COVID‐19 (Table 2).

**TABLE 2:** In‐group and between‐group comparison (tinnitus frequency, tinnitus severity, MMS, RI, VAS [severity and annoyance]).

	Group 1/a mean ± SD	Group 1/b mean ± SD	*p*	95% CI	Group 2/a mean ± SD	Group 2/b mean ± SD	*p*	95% CI	Inter group/*p*	
PTA	18.20 ± 3.58	18.93 ± 3.76	0.094	[−1.60/0.14]	17.86 ± 3.64	18.40 ± 3.48	0.056	[−1.08/0.01]	0.338	0.120
Tinnitus frequency (Hz)	9.53 ± 2.29‐	9.26 ± 1.94	0.164	[−0.123/0.65]	8.26 ± 3.08	8.66 ± 3.17	0.655	[−2.28/1.48]	0.212	0.538
Tinnitus severity (dB)	59.13 ± 17.18	58.13 ± 16.83	0.910	[−2.98/2.98]	60.40 ± 12.94	69.73 ± 10.40	< 0.001**	[−12.54/−6.12]	0.821	0.047*
MMS (dB SL)	68.13 ± 18.60	67.60 ± 18.36	0.495	[−1.09/2.16]	68.86 ± 12.09	74.66 ± 9.28	0.013*	[−10.19/−1.40]	0.899	0.194
RI	1.46 ± 0.83	1.40 ± 0.83	0.836	[−0.61/0.74]	1.00 ± 0.84	1.26 ± 0.96	0.801	[−0.83/0.30]	0.821	0.899
VAS (severity)	5.60 ± 1.40	5.93 ± 0.96	0.136	[−0.78/0.11]	4.86 ± 1.06	7.06 ± 1.16	< 0.001**	[−2.72/−1.67]	0.118	0.007**
VAS (annoyance)	5.33 ± 1.58	5.86 ± 0.99	0.178	[−1.34/0.27]	5.26 ± 2.76	7.00 ± 1.19	0.006**	[−2.86/−0.59]	0.936	0.009**

*Note*: Data were presented as mean ± standard deviation and CI (confidence interval). Within‐group changes were presented as *p < 0.05 and **p < 0.01.

Abbreviations: Group 1, COVID‐19 (−); Group 2, COVID‐19 (+); Group 1/a, before pandemic; Group 1/b, after pandemic, Group 2/a, before pandemic; Group2/b, after pandemic; MMI, minimal masking level; PTA, pure tone audiometry; RI, residual inhibition; VAS, Visual Analog Scale.

**FIGURE 1:**
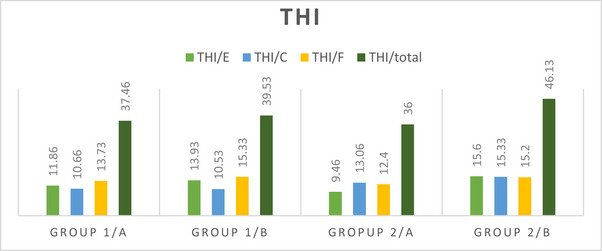
In‐group and between‐group comparison (THI). Group 1/a, before pandemic; Group 1/b, after pandemic; Group 2, COVID‐19 (+); Group 2/a, before pandemic; Group 2/b, after pandemic; Group1, COVID‐19 (−); THI/C, THI catastrophic; THI/E, THI emotional; THI/F, THI functional.

THI emotional subscale scores of Group 1 worsened after COVID‐19, compared to before COVID‐19 (*p* < 0.001) (Table 3; Figure 1). Similarly, after the pandemic, compared to before the pandemic, the total THI score and all subscale scores of Group 2 were worse (*p* < 0.001) (Table 3; Figure 1).

**TABLE 3:** In‐group and between‐group comparison (THI).

Group 1					Group 2				Intergroup	
THI	Group 1/a mean ± SD	Group1/b mean ± SD	*p*	95% CI [lower/upper]	Group 2/a mean ± SD	Group 2/b mean ± SD	*p*	95% CI [lower/upper]	*p*/a	*p*/b
THI/E	11.86 ± 3.33	13.93 ± 4.39	**0.017**	[−2.83/7.70]	9.46 ± 3.58	15.60 ± 2.16	**< 0.001**	[−8.51/−3.74]	0.068	**0.044**
THI/C	10.66 ± 3.17	10.53 ± 4.10	0.827	[−1.15/1.42]	13.06 ± 3.28	15.33 ± 3.51	**0.006**	[−3.76/−0.76]	0.051	**0.002**
THI/F	13.73 ± 4.13	15.33 ± 4.70	0.104	[−3.57/0.37]	12.40 ± 4.15	15.20 ± 5.64	**0.037**	[−5.40/−0.19]	0.386	0.944
THI total	37.46 ± 9.11	39.53 ± 8.95	0.495	[−6.78/4.65]	36.00 ± 8.24	46.13 ± 6.43	**< 0.001**	[−14.43/−5.83]	0.648	**0.013**

*Note*: Data were presented as mean ± standard deviation and CI (confidence interval).

Abbreviations: Group 1/a, before pandemic; Group 1/b, after pandemic; Group 1: COVID‐19 (−); Group 2/a, before pandemic; Group 2/b, after pandemic; Group 2: COVID‐19 (+); THI, Tinnitus Handicap Inventory—consists of 24 items—that are grouped in three subscales or domains; THI/C, THI catastrophic; THI/E, THI emotional; THI/F, THI functional.

THI emotional (*p* = 0.044), THI catastrophic (*p* = 0.002), and the THI total score (*p* = 0.013) in patients who had COVID‐19 were worse than in patients who did not have COVID‐19 (Table 3).

**FIGURE 2:**
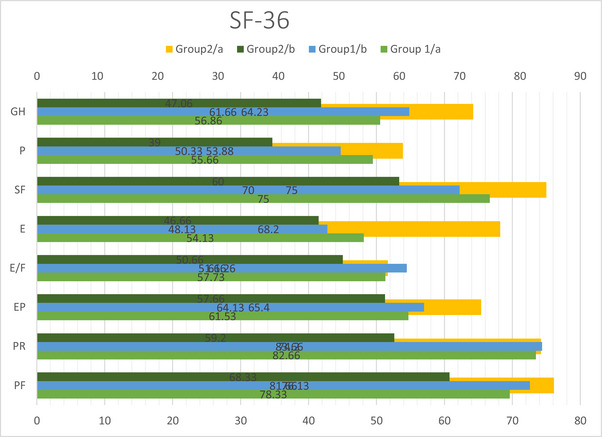
In‐group and between‐group comparison (SF‐36). Within‐group changes were presented as *p < 0.05 and **p < 0.01. E, emotional well‐being; E/P, energy/fatigue; EP, role limitations due to emotional problems; GH, general health; Group 1, COVID‐19 (−); Group 1/a, before pandemic; Group 2, COVID‐19 (+); Group 2/a, before pandemic; Group 2/b, after pandemic; Group1/b, after pandemic; P, pain; PF, physical functioning; PR, role limitations due to physical health; SF, social functioning.

Physical functioning (CI: 1.94*–*13.52, *p* = 0.044), emotional problems limiting roles (1.94*–*13.52, *p* = 0.012), emotional well‐being (5.17*–*37.89, *p* = 0.014), pain (13.57*–*36.20, *p* < 0.01), and GH scores (9.92*–*24.40, *p* < 0.001) of Group 2 worsened after COVID‐19, compared to before COVID‐19 (Table 4; Figure 2). Emotional well‐being (−10.71 to −1.28, *p* = 0.016) and GH scores of Group 1 worsened after COVID‐19, compared to before COVID‐19 (−7.70 to 1.89, *p* = 0.003) (Table 4; Figure 2).

**TABLE 4:** In‐group and between‐group comparison (SF‐36).

	Group 1				Group 2				Intergroups	
SF‐36	Group 1/a mean ± SD	Group 1/b mean ± SD	*p*	95% CI [lower/upper]	Group 2/a mean ± SD	Group 2/b mean ± SD	*p*	95% CI [lower/upper]	*p*/a	*p*/b
PF	78.33 ± 14.09	81.66 ± 12.77	0.116	[−7.60/0.93]	76.13 ± 22.97	68.33 ± 21.19	0.253	[−6.23/21.83]	0.754	0.106
PR	82.66 ± 24.04	83.66 ± 22.39	0.864	[−13.30/11.30]	74.20 ± 27.00	59.20 ± 28.84	0.076	[−1.18/31.81]	0.372	**0.044**
EP	61.53 ± 11.94	64.13 ± 14.83	0.431	[−9.48/4.28]	65.40 ± 17.38	57.66 ± 16.39	**0.012**	[1.94/13.52]	0.484	0.267
E/F	57.73 ± 19.50	61.26 ± 14.83	0.140	[−8.37/1.31]	51.66 ± 25.40	50.66 ± 25.27	0.837	[−9.26/11.26]	0.469	0.184
E	54.13 ± 19.40	48.13 ± 22.58	**0.016**	[−10.71/−1.28]	68.20 ± 27.78	46.66 ± 23.34	**0.014**	[5.17/37.89]	**0.048**	0.289
SF	75.00 (25–100)	70 (25–100)	0.709	[−1.37/12.17]	75 (25–100)	60 (0–100)	0.462	[−4.25/15.92]	0.700	0.453
P	55.66 ± 24.48	50.33 ± 25.89	0.299	[−5.27/15.94]	53.88 ± 21.98	39.00 ± 20.80	**< 0.001**	[13.57/36.20]	0.836	**0.019**
GH	56.86 ± 14.19	61.66 ± 14.66	**0.003**	[−7.70/1.89]	64.23 ± 17.98	47.06 ± 13.76	**< 0.001**	[9.92/24.40]	0.224	**0.009**

*Note*: Data were presented as mean ± standard deviation and CI (confidence interval). The SF‐36 consists of 36 items—that are grouped in eight subscales or domains. Within‐group changes were presented as *p < 0.05 and **p < 0.01.

Abbreviations: E, emotional well‐being; E/P, energy/fatigue; EP, role limitations due to emotional problems; GH, general health; Group 1, COVID‐19 (−); Group 1/a, before pandemic; Group 1/b, after pandemic; Group 2, COVID‐19 (+); Group2/a, before pandemic; Group2/b, after pandemic; P, pain; PF, physical functioning; PR, role limitations due to physical health; SF, social functioning.

## 4 Discussion

This research study investigated the impact of COVID‐19 on the severity of tinnitus and on the quality of life of individuals with tinnitus. It was shown to have affected both tinnitus severity and the quality of life of individuals with tinnitus who had been infected with COVID‐19. At the same time, tinnitus had psychosomatic and quality‐of‐life effects on individuals with tinnitus who had not been infected with COVID‐19, but the effects were not as significant as in the infected group. A total of 30 individuals with tinnitus, both with and without COVID‐19 infection, were evaluated. The balanced distribution of patient characteristics in both groups provides an appropriate basis for assessing changes in tinnitus patterns associated with COVID‐19. Despite the limited number of participants, this balance increases the reliability and generalizability of our findings.

We repeated the audiological and tinnitus psychoacoustic–psychosomatic evaluations, which had been evaluated before the COVID‐19 pandemic and after the outbreak of the pandemic, and compared the scores both within and between groups. Our study categorized the severity of individuals with COVID‐19, with most individuals experiencing mild (*n* = 8) or moderate (*n* = 5) illness and only a small number of individuals experiencing severe (*n* = 2) COVID‐19. However, not all individuals required hospitalization, suggesting that the study population consisted of relatively less severe cases.

In recent years, the impact of the COVID‐19 pandemic on tinnitus has become a subject of growing interest. Several studies have examined the relationship between COVID‐19 and tinnitus, often highlighting the role of anxiety and stress. For instance, Xia et al. (2021) reported that COVID‐19‐associated anxiety significantly exacerbates tinnitus symptoms, using the THI to assess the severity of tinnitus in patients. Similarly, Chatterjee and Gore (2021) compared tinnitus management strategies during the COVID‐19 pandemic, again employing the THI to evaluate treatment outcomes. Fioretti et al. (2022) found that the COVID‐19 lockdown had a substantial impact on individuals with chronic tinnitus, using the THI to assess changes in tinnitus severity over time. Erinc et al. (2022) explored the long‐term effects of COVID‐19 on tinnitus, revealing persistent symptom exacerbation in some patients. While these studies have contributed valuable insights, there remains a gap in understanding the specific mechanisms by which COVID‐19 affects tinnitus, particularly in terms of changes in tinnitus characteristics and the role of hearing loss.

This study aims to address these gaps by investigating the impact of COVID‐19 infection on tinnitus symptoms using the THI, with a specific focus on pre‐ and post‐pandemic comparisons. Unlike previous studies, which primarily focused on anxiety and stress, our study explores both the psychological and auditory aspects of tinnitus, examining not only the severity of tinnitus but also changes in hearing thresholds post‐pandemic.

Studies since the onset of the pandemic have indicated that hearing impairment/disorder (e.g., tinnitus and hearing loss) may be associated with COVID‐19 (Chatterjee and Gore 2021; Fioretti et al. 2022). The mechanism of auditory disorders associated with COVID‐19 is linked to either the viral infiltration of cranial nerves or an immune‐mediated response (Erinc et al. 2022; Mui et al. 2023). It is believed that the COVID‐19 virus can affect the function of cochlear outer hair cells. In addition, other proposed mechanisms suggest that COVID‐19 infection, along with serotonin release and blood coagulation, may contribute to the emergence of tinnitus by activating platelets (E. Beukes et al. 2021; Can et al. 2023). When comparing the average hearing thresholds before and after the pandemic, no statistically significant difference was observed; however, a decline in hearing thresholds was noted post‐pandemic. This finding aligns with studies in the literature addressing the potential effects of COVID‐19 on hearing loss (Almufarrij and Munro 2021). This highlights the need for further investigation into hearing loss within a broader research context.

Almufarrij and Munro (2021) suggested an estimated prevalence of tinnitus at 14.8%, and another study reported that 10% of the population had tinnitus.

Some studies, such as those by Almufarrij and Munro (2021), have reported an association between an increase in tinnitus complaints and COVID‐19 and its consequences. However, further research is needed to confirm these findings (E. Beukes et al. 2021).

It has been reported that patients may develop hearing loss related to tinnitus after COVID‐19 infection, but it has also been observed that some patients developed tinnitus without their hearing being affected (Erinc et al. 2022). To investigate the effect of COVID‐19 on tinnitus alone, we have included subjects with normal hearing in the study. In one study, patients reporting tinnitus after a diagnosis of COVID‐19 had a mean VAS score of 5, indicating a common moderate level of complaints among patients (Viola et al. 2020).

Similar to the literature, in this study, it was observed that severity of tinnitus and MML increased in the psychoacoustic evaluations of tinnitus occurring after COVID‐19 infection. We found that this increase was reflected in the psychosomatic scores. When we compared the pre‐ and post‐pandemic scores, the increase in the VAS severity and discomfort levels of Group 2 (COVID‐19 positive) as reflected in our scores was significant. In Group 1, however, no significant difference was found.

Because COVID‐19 is thought to affect cochlear outer hair cell function (E. Beukes et al. 2021; Swain et al. 2022) and because of its similarities with the proposed pathophysiology of tinnitus (Makar 2021), we hypothesized that COVID‐19 was the cause of increased tinnitus severity in tinnitus patients with COVID‐19. In addition, when VAS severity and degree of discomfort scores before and after the pandemic were compared in Group 1, no significant difference was obtained, although we did find an increase. It is important to note that some of the negative effects of the pandemic are fear, stress, and anxiety, and experiencing these effects can negatively affect many existing health problems.

Important research (Aydogan, Satekin, Ocak, et al. 2022; Mazurek et al. 2012) has been conducted on the molecular relationship between anxiety, stress, and tinnitus. In these studies, limbic and prefrontal areas were associated with emotion, emotional states (such as stress, sadness, and anxiety), and tinnitus. It has been suggested that in many people with tinnitus, these distressing emotions may contribute to the perception of tinnitus (Jastreboff and Hazell 2008). We think that the increase was due to the fact that emotions, such as stress, anxiety, fear, and worry, negatively affected the patients' perception of tinnitus amid the increased anxiety and stress during the pandemic.

Xia et al. (2020) reported a significantly‐severe tinnitus during the pandemic (40/100 for THI in 99 patients), than before the pandemic (34/100 for THI in 89 patients). The study found that a relationship was found between the COVID‐19 pandemic and increased anxiety and THI. While an increase in psychological stress was observed as an effect of the pandemic, it was emphasized that this situation caused an increase in THI and tinnitus severity.

Freni et al. (2020) reported a THI score of 6.6 ± 12.1 and the initiation or worsening of tinnitus in 10 patients due to COVID‐19. In a study (Kartal and Kılıc 2023), the mean VAS score for severity, frequency/duration, and discomfort of the participants with tinnitus after COVID‐19 was 4.37 ± 2.37, while the mean THI score was 49.56 ± 9.81. Consistent with the literature, in our study, VAS scores for severity and degree of discomfort, THI total scores (for Group 2, 46.13 ± 6.43), and the scores for the emotional, catastrophic, and functional subdomains of the THI significantly increased when compared with the pre‐pandemic scores in Group 2. In Group 1, there was a worsening of VAS severity and degree of discomfort and THI catastrophic and functional scores after the pandemic, but there was no significant difference when scores before and after the pandemic were compared. There was a noticeable difference only in the emotional subdomain of the THI. We assume that this was due to the negative impact of the pandemic on humans.

The COVID‐19 pandemic disrupted our entire lives. Restrictive measures that changed the way we socialized, interacted, received education, and accessed health care were imposed to reduce virus transmission. These measures have had a negative impact on quality of life and increased stress, although they have reduced the transmission of the virus (Janiri et al. 2021). An up‐to‐date systematic review of trials dealing with COVID‐19 and audio‐vestibular symptoms indicated the negative impact these measures had on well‐being (Almufarrij and Munro 2021; Viola et al. 2020).

A study conducted on the development of significant mental stress due to the COVID‐19 pandemic reported that anxiety associated with COVID‐19 increased tinnitus severity (Xia et al. 2021). The perception of tinnitus is known to be negatively affected by emotions such as stress, anxiety, and fear. Moreover, there are studies (Aydogan, Satekin, Ocak, et al. 2022; Xia et al. 2020; Gurses and Cildir 2022) on the effects of stress, anxiety, and social isolation caused by the pandemic on the perception of tinnitus and quality of life. However, there are limited studies on the severity of tinnitus in individuals with COVID‐19 infection. With this study, we emphasized both the effects of COVID‐19 infection on tinnitus severity and quality of life and the negative effects of the pandemic.

Studies (Aydogan, Satekin, Uyar, et al. 2022; E. W. Beukes et al. 2020) have shown that tinnitus negatively affects the quality of life. Another study reported that the THI and SF‐36 scores of individuals with tinnitus worsened during the COVID‐19 pandemic and that the pandemic was a source of stress. In our study, the SF‐36 was used to evaluate the quality of life. Our study sought to determine the effects of the COVID‐19 pandemic on tinnitus and quality of life. We evaluated the quality of life before the pandemic in individuals with tinnitus and after the pandemic began, we evaluated individuals with tinnitus both with and without a COVID‐19 diagnosis. We found that the quality of life of Group 2 (COVID‐19 positive) was more negatively affected. At the same time, the quality of life of Group 1 (uninfected with COVID‐19) was negatively affected but less dramatically than Group 2. There was a significant difference in the before and after the pandemic scores in terms of the SF‐36 total score and its subdomains in Group 2. We concluded that one of the negative effects of the pandemic was the decrease in the quality of life. At the same time, we assumed that Group 2 was more negatively affected due to the complications of the COVID‐19 infection.

### 4.1 Conclusion and Limitations

This study demonstrates that COVID‐19 negatively affected both the quality of life and the severity of tinnitus in individuals with tinnitus, with more pronounced effects observed in the group infected with COVID‐19. The stress, anxiety, and social isolation caused by the pandemic are likely contributors to these negative outcomes. In addition, the greater degree of impact in the COVID‐19‐infected group may be linked to the virus's effects on multiple body systems, potentially exacerbating tinnitus symptoms.

However, this study has several limitations that should be noted. The small sample size may limit the generalizability of the findings and reduce the statistical power to detect subtle effects. Furthermore, the reliance on self‐reported data for tinnitus severity and associated symptoms may introduce recall bias and subjective variability. The challenges posed by the pandemic, such as patients' reluctance to attend follow‐up evaluations and logistical constraints, also limited the sample size and data collection. Finally, the cross‐sectional nature of the study prevents the establishment of causal relationships between COVID‐19 and tinnitus severity.

In future pandemics or similar public health crises, the implementation of telehealth services, tinnitus management counseling, and strategies to address stress and anxiety may mitigate the impact of such events on individuals with tinnitus. We also recommend further longitudinal studies with larger and more representative samples to better understand the relationship between systemic infections, psychological stressors, and tinnitus outcomes.

## Author Contributions

**Zehra Aydogan**: conceptualization, methodology, supervision, resources, validation, writing–original draft. **Mehmet Can**: conceptualization, investigation, funding acquisition, data curation. **Emre Soylemez**: funding acquisition, writing–review and editing, formal analysis, software, supervision, resources, methodology, validation. **Kursad Karakoc**: conceptualization, investigation, funding acquisition, writing–original draft. **Zahide Ciler Buyukatalay**: methodology, validation, visualization, writing–review and editing. **Suna Tokgoz Yilmaz**: software, formal analysis, project administration, data curation, supervision, resources.

## Conflicts of Interest

The authors declare no conflicts of interest.

### Peer Review

The peer review history for this article is available at https://publons.com/publon/10.1002/brb3.70317.

## Data Availability

The data that support the findings of this study are available on request from the corresponding author. The data are not publicly available due to privacy or ethical restrictions.
